# Clinical Outcomes of Severe COVID-19 Patients Admitted to an Intermediate Respiratory Care Unit

**DOI:** 10.3389/fmed.2021.711027

**Published:** 2021-07-01

**Authors:** Guillermo Suarez-Cuartin, Merce Gasa, Guadalupe Bermudo, Yolanda Ruiz, Marta Hernandez-Argudo, Alfredo Marin, Pere Trias-Sabria, Ana Cordoba, Ester Cuevas, Mikel Sarasate, Albert Ariza, Joan Sabater, Nuria Romero, Cristina Subirana, Maria Molina-Molina, Salud Santos

**Affiliations:** ^1^Respiratory Department, Bellvitge University Hospital, Bellvitge Biomedical Research Institute (IDIBELL), L'Hospitalet de Llobregat, Spain; ^2^Cardiology Department, Bellvitge University Hospital, Bellvitge Biomedical Research Institute (IDIBELL), L'Hospitalet de Llobregat, Spain; ^3^Critical Care Department, Bellvitge University Hospital, Bellvitge Biomedical Research Institute (IDIBELL), L'Hospitalet de Llobregat, Spain

**Keywords:** COVID-19, pneumonia, mortality, IMCU, ICU, non-invasive ventilation, high-flow nasal cannula, intermediate care unit

## Abstract

**Introduction:** Many severe COVID-19 patients require respiratory support and monitoring. An intermediate respiratory care unit (IMCU) may be a valuable element for optimizing patient care and limited health-care resources management. We aim to assess the clinical outcomes of severe COVID-19 patients admitted to an IMCU.

**Methods:** Observational, retrospective study including patients admitted to the IMCU due to COVID-19 pneumonia during the months of March and April 2020. Patients were stratified based on their requirement of transfer to the intensive care unit (ICU) and on survival status at the end of follow-up. A multivariable Cox proportional hazards method was used to assess risk factors associated with mortality.

**Results:** A total of 253 patients were included. Of them, 68% were male and median age was 65 years (IQR 18 years). Ninety-two patients (36.4%) required ICU transfer. Patients transferred to the ICU had a higher mortality rate (44.6 vs. 24.2%; *p* < 0.001). Multivariable proportional hazards model showed that age ≥65 years (HR 4.14; 95%CI 2.31–7.42; *p* < 0.001); chronic respiratory conditions (HR 2.34; 95%CI 1.38–3.99; *p* = 0.002) and chronic kidney disease (HR 2.96; 95%CI 1.61–5.43; *p* < 0.001) were independently associated with mortality. High-dose systemic corticosteroids followed by progressive dose tapering showed a lower risk of death (HR 0.15; 95%CI 0.06–0.40; *p* < 0.001).

**Conclusions:** IMCU may be a useful tool for the multidisciplinary management of severe COVID-19 patients requiring respiratory support and non-invasive monitoring, therefore reducing ICU burden. Older age and chronic respiratory or renal conditions are associated with worse clinical outcomes, while treatment with systemic corticosteroids may have a protective effect on mortality.

## Introduction

Coronavirus disease 2019 (COVID-19) is a respiratory condition caused by severe acute respiratory syndrome-coronavirus-2 (SARS-CoV-2) (1). Patients with COVID-19 may become severely ill and require hospital admission, with estimated hospitalization rates of 1 to 18%, increasing with older age (2). Current recommendations state that patients with COVID-19 related acute respiratory failure should be monitored, and support with high-flow nasal cannula (HFNC) oxygen therapy or non-invasive ventilation (NIV) should be considered when conventional oxygen therapy fails (3). In this regard, during the months of March and April of 2020, the COVID-19 pandemic conditioned a significant increase in healthcare burden across Europe, as 17 to 32% of admitted patients required critical care management (4–7). The intensive care unit (ICU) beds and invasive mechanical ventilators achieved their limits of occupation, hence non-invasive supportive care was a valuable option for maintaining respiratory conditions. Therefore, a proper healthcare resource management was necessary to warrant an adequate patient care.

Intermediate respiratory care units (IMCU) are a useful resource for the management of complex patients that do not require admission to the ICU, invasive mechanical ventilation or invasive monitoring (8). IMCU can function as a space for management escalation and de-escalation between the general ward and the ICU, especially when patient monitoring is needed and/or when respiratory support with HFNC or NIV is required (8–10). Benefits of IMCU include reducing ICU admission time and increasing ICU bed capacity, as well as lowering mortality and health care costs (8, 10, 11). Although the role of the ICU is well-known, there is scarce data regarding the outcomes of severe COVID-19 patients managed in an IMCU during the pandemic.

To this date, there have been more than 158 million reported cases worldwide, with over 3 million deaths (12). Mortality is variable among hospitalized patients with COVID-19 pneumonia. Studies from Chinese cohorts estimate a hospital mortality of 4 to 28% (4, 5, 13). Furthermore, a recent study from the United Kingdom showed an overall mortality of 26% in admitted patients (7). Most of these deaths were related to sepsis, respiratory failure, acute respiratory distress syndrome (ARDS) and heart failure (4). Moreover, mortality rates of patients in critical care are higher, ranging from 26 to 32% (7, 14), including ICU and IMCU. Nevertheless, the specific mortality of COVID-19 patients admitted to an IMCU has not been widely studied.

We aim to evaluate the outcomes of severely ill COVID-19 patients requiring monitoring and/or non-invasive respiratory support admitted to an IMCU, and to identify clinical factors that may have led to these results.

## Methods

### Study Design

**Figure 1:**
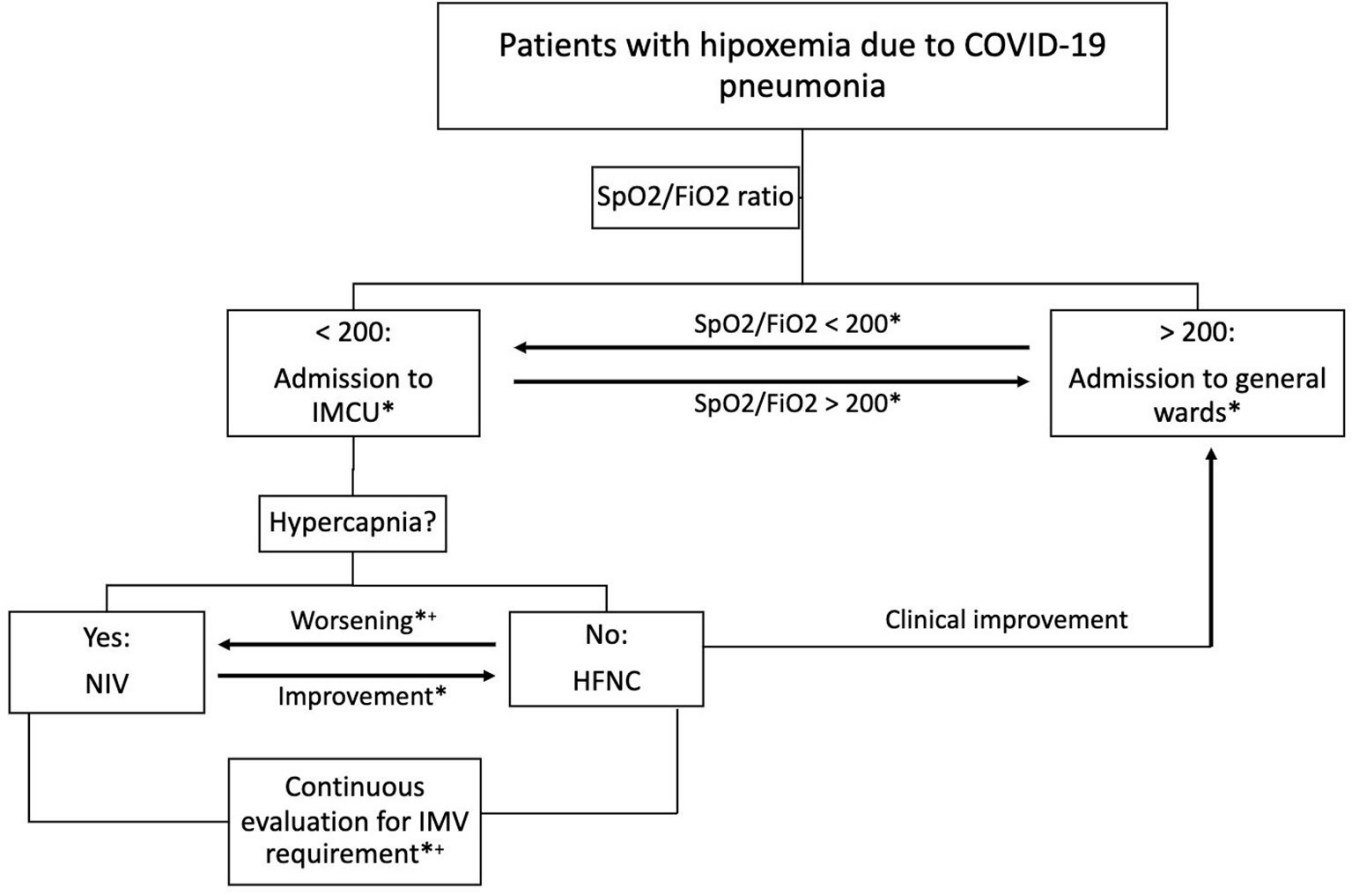
Algorithm of multidisciplinary patient management. Clinical worsening is defined as SpO2/FiO2 <100 and/or tachypnea >30 breaths per minute. Clinical improvement is defined as SpO2/FiO2 >200 and respiratory rate <30 breaths per minute. ^*^Daily multidisciplinary team assessment; ^+^Transfer to ICU if IMV and/or vasoactive drugs are required. COVID-19, coronavirus disease-2019; SpO2/FiO2, oxygen saturation to fraction of inspired oxygen ratio; IMCU, intermediate respiratory care unit; ICU, intensive care unit; NIV, non-invasive ventilation; HFNC, high-flow nasal cannula; IMV, invasive mechanical ventilation.

An observational and retrospective study was performed on consecutive patients admitted to the IMCU of a tertiary care hospital in Barcelona (Spain) throughout the months of March and April 2020. The final date of follow-up was June 28, 2020. Study protocol was approved by the local ethics committee (No. PR260/20). Inclusion criterion was admission to IMCU due to respiratory failure related to COVID-19 pneumonia requiring non-invasive monitoring and/or non-invasive respiratory support. Patients were diagnosed with a positive polymerase chain reaction for SARS-CoV2 from nasopharyngeal swab and the presence of pulmonary opacities on chest X-ray. According to local protocol, IMCU admission was limited to subjects with an oxygen saturation (SpO2) to inspired oxygen fraction (FiO2) ratio lower than 200 but not expected to require immediate support with invasive mechanical ventilation. Subjects were admitted directly from the emergency department or transferred from a regional hospital or general wards due to clinical impairment. Exclusion criteria were: recent admission to the ICU, as these patients had already shown clinical improvement, subjects with COVID-19 pneumonia not requiring oxygen support through HFNC or NIV, and respiratory failure due to any etiology other than COVID-19. Figure 1 shows the algorithm of multidisciplinary patient management including specialists in respiratory medicine, critical care and internal medicine.

### Data Collection and Analysis

Demographic, clinical, radiological and laboratory data were collected from electronic medical records for all patients at the time of IMCU admission. All participants were treated according to hospital protocols. Systemic corticosteroid therapy was divided into four categories depending on dose and administration route, as patients were treated before preliminary results from the RECOVERY trial (15): (a) no corticosteroid treatment, (b) and (c) intravenous (IV) bolus of 1–2 mg/Kg/day methylprednisolone or its equivalent dexamethasone dose for 3 days, followed or not by oral prednisone starting from 0.5 mg/Kg/day, tapering the dose over 7 to 10 days; and (d) only oral prednisolone in doses lower than 0.5 mg/Kg/day for 7 to 10 days. Treatment schemes were chosen depending on clinical and radiological severity, where more severe individuals received longer treatments and higher doses. Patients were categorized depending on survival status and ICU transfer requirement during hospitalization. ICU admission criteria included cardiopulmonary arrest, sudden fall in level of consciousness, invasive ventilation requirement and shock requiring support with vasoactive drugs. The decision of whether or not to transfer a patient to the ICU was always made by a multidisciplinary team including pulmonologists and intensive care physicians. For the survival analysis, clinical and laboratory features were studied using criteria for ARDS (16) and cut-off values identified in severe cases from previous studies (4, 5, 17–19).

### Statistical Analysis

Frequency and percentages were used to present categorical data, and chi-squared test or Fisher's exact test were used to evaluate their differences. Continuous variables are expressed as mean and standard deviation (SD) for normally distributed variables or median and interquartile range (IQR) otherwise. ANOVA and Student's *t* test or their corresponding non-parametrical tests (Kruskal–Wallis and Mann–Whitney *U* tests, respectively) were used to evaluate their differences, when required. Kaplan–Meier curves were used for the survival analysis. In order to identify factors associated with mortality, a multivariable Cox proportional hazards analysis was performed including significant variables from univariate analysis. A *p*-value < 0.05 was considered statistically significant. Data were analyzed using R (software version 3.6.2).

## Results

### Patient Description

A total of 291 patients were admitted to the IMCU during the months of March and April of 2020 due to COVID-19 pneumonia. After excluding 38 patients that were previously treated in the ICU, 253 patients were finally included. Of them, 68% were male and median age was 65 years (IQR 18 years). The most frequent comorbidities were hypertension (50.2%), dyslipidemia (47.8%) and diabetes mellitus (29.6%). Demographic and clinical characteristics of included patients at admission to IMCU are described in Table 1.

**Table 1:** Characteristics of all patients admitted to the respiratory intermediate care unit, and according to requirement of transfer to the ICU.

	**Total (*N* = 253)**	**No ICU admission (*N* = 161)**	**ICU admission (*N* = 92)**	***P*-value**
Male, *n* (%)	172 (67.9%)	104 (64.6%)	68 (73.9%)	0.165
Age in years, median (IQR)	65 (18)	66 (19)	63 (15.3)	0.072
**Comorbidities**				
Hypertension, *n* (%)	127 (50.2%)	85 (52.8%)	42 (45.7%)	0.336
Diabetes, *n* (%)	75 (29.6%)	44 (27.3%)	31 (33.7%)	0.356
Dyslipidemia, *n* (%)	121 (47.8%)	75 (46.6%)	46 (50%)	0.695
Obesity, *n* (%)	63 (24.9%)	40 (24.9%)	23 (25%)	>0.999
Cardiovascular disease, *n* (%)	27 (10.7%)	19 (11.8%)	8 (8.7%)	0.577
**Chronic respiratory disease**, ***n*** **(%)**				
Asthma	14 (5.5%)	8 (4.9%)	6 (6.5%)	0.789
COPD	17 (6.7%)	13 (8.1%)	4 (4.3%)	
Interstitial lung disease	6 (2.4%)	3 (1.9%)	3 (3.3%)	
Bronchiectasis	1 (0.4%)	1 (0.6%)	0	
OSAS	15 (5.9%)	9 (5.6%)	6 (6.5%)	
History of malignancy, *n* (%)	37 (14.6%)	24 (14.9%)	13 (14.1%)	>0.999
Chronic liver disease, *n* (%)	22 (8.7%)	18 (11.2%)	4 (4.4%)	0.105
Chronic kidney disease, *n* (%)	27 (10.7%)	19 (11.8%)	8 (8.7%)	0.577
Immunosuppression, *n* (%)	13 (5.1%)	10 (6.2%)	3 (3.3%)	0.386
**Symptoms**				
Dyspnea, *n* (%)	149 (58.9%)	86 (53.4%)	63 (68.5%)	0.027^*^
Cough, *n* (%)	189 (74.7%)	117 (72.7%)	72 (78.3%)	0.405
Fever, *n* (%)	211 (83.4%)	131 (81.4%)	80 (86.9%)	0.330
Myalgias, *n* (%)	71 (28.1%)	49 (30.4%)	22 (23.9%)	0.334
Diarrhea, *n* (%)	64 (25.3%)	38 (23.6%)	26 (28.3%)	0.503
Nausea, *n* (%)	22 (8.7%)	12 (7.5%)	10 (10.9%)	0.487
Days from symptom onset to hospital admission), median (IQR)	8 (5)	8 (5)	8 (5)	0.821
Days from symptom onset to IMCU admission), median (IQR)	10 (7)	10 (7)	9 (7.3)	0.176
**Chest X-ray on admission**				
Bilateral opacities, *n* (%)	149 (58.9%)	143 (90.5%)	89 (96.7%)	0.113
Peripheral distribution of opacities, *n* (%)	189 (74.7%)	96 (60.8%)	48 (52.2%)	0.233
**Laboratory blood tests**				
Leukocyte count (× 10^9^/L), median (IQR)	8.60 (5.6)	7.80 (4.8)	10.15 (6.5)	<0.001^*^
Lymphocyte count (× 10^9^/L), median (IQR)	0.68 (0.6)	0.73 (0.6)	0.64 (0.6)	0.039^*^
Lactate dehydrogenase (U/L), median (IQR)	418 (221)	398 (220.5)	446 (237)	0.005^*^
C-Reactive protein (mg/L), median (IQR)	137 (177)	108 (148)	179.50 (188)	<0.001^*^
Ferritin (μg/L), median (IQR)	1,443 (1,337)	1,479 (1,426)	1,410 (1,242.8)	0.829
D-dimer (μg/L), median (IQR)	531 (814.3)	506.50 (821.3)	599.50 (757.5)	0.446
**Treatment**				
Lopinavir/Ritonavir, *n* (%)	202 (79.8%)	126 (78.3%)	76 (82.6%)	0.505
Remdesivir, *n* (%)	11 (4.4%)	2 (1.2%)	9 (9.8%)	0.004^*^
Hydroxychloroquine, n (%)	243 (96.1%)	151 (93.8%)	92 (100%)	0.035^*^
Tocilizumab, *n* (%)	124 (49%)	73 (45.3%)	51 (55.4%)	0.157
**Systemic corticosteroids**, ***n*** **(%)**				
Intravenous bolus	114 (45.1%)	67 (41.6%)	47 (51.1%)	0.022^*^
Intravenous bolus + oral tapering regimen	52 (20.6%)	28 (17.4%)	24 (26.1%)	
Oral tapering regimen	4 (1.6%)	3 (1.9%)	1 (1.1%)	
SpO2/FiO2 ratio, median (IQR)	132.90 (52.5)	137.10 (74.3)	118.80 (37.1)	<0.001^*^
**Respiratory support**				
High-flow oxygen, *n* (%)	165 (65.2%)	88 (54.7%)	77 (83.7%)	<0.001^*^
Non-invasive ventilation, *n* (%)	133 (52.6%)	58 (36%)	75 (81.5%)	<0.001^*^
Invasive mechanical ventilation, *n* (%)	82 (32.4%)	0	82 (89.1%)	<0.001^*^

*ICU, intensive care unit; IQR, interquartile range; COPD, chronic obstructive pulmonary disease; OSAS, obstructive sleep apnea syndrome; SpO2, oxygen saturation; FiO2, fraction of inspired oxygen; IMCU, Intermediate care unit.*

* *Indicates significant p-value < 0.05*.

### Clinical Outcomes

Ninety-two patients (36.4%) required transfer to the ICU. There were no significant differences in age, gender or comorbidities between ICU and non-ICU groups. However, patients requiring ICU management had higher systemic inflammatory markers and a significant lower SpO2/FiO2 ratio on admission. A comparison of patient characteristics between those that were admitted to the ICU and those who did not, is shown in Table 1.

A higher proportion of patients received home discharge in the IMCU group compared to those that required transfer to ICU (50.3 vs. 22.8%, respectively; *p* < 0.001). However, a similar proportion of subjects needed admission to socio-health centers or transfer to their regional hospital for convalescence. Six patients of the ICU group were still hospitalized, while none of the IMCU subjects were in the hospital at the end of follow-up. A comparison of clinical outcomes between groups is presented in Table 2.

**Table 2:** Clinical outcomes of all patients admitted to the respiratory intermediate care unit, and according to requirement of transfer to the ICU.

	**Total (*N* = 253)**	**No ICU admission (*N* = 161)**	**ICU admission (*N* = 92)**	***P*-value**
Home discharge, *n* (%)	103 (40.7%)	81 (50.3%)	22 (23.9%)	<0.001^*^
Socio-health center transfer, *n* (%)	70 (27.7%)	41 (25.5%)	29 (31.5%)	>0.999
Deaths during admission, *n* (%)	80 (31.6%)	39 (24.2%)	41 (44.6%)	0.001^*^
Length of IMCU stay in days, median (IQR)	6 (7)	7 (6.3)	4 (5.3)	<0.001^*^
Length of hospital stay in days, median (IQR)	16 (19)	13 (10.3)	31 (31)	<0.001^*^

*IQR, interquartile range; ICU, intensive care unit; IMCU, intermediate care unit.*

* *Indicates significant p-value < 0.05*.

### Mortality

Eighty patients (31.6%) died during hospitalization. Main causes of death were ARDS and septic shock. Patients requiring transfer to the ICU had a higher mortality rate (44.6 vs. 24.2%; *p* < 0.001). When comparing survivors and non-survivors, a significant difference was observed regarding age (median 61 years, IQR 17 vs. median 72 years, IQR 10.3, respectively; *p* < 0.001). Non-survivors had a higher proportion of comorbidities such as dyslipidemia, chronic respiratory diseases and chronic kidney disease. Furthermore, these patients had higher blood leukocyte counts, serum lactate dehydrogenase (LDH), C-reactive protein and D-dimer, and lower blood lymphocyte counts on admission to the IMCU (Table 3).

**Table 3:** Patient characteristics regarding in-hospital mortality.

	**Survivors (*N* = 173)**	**Non-survivors (*N* = 80)**	***P*-value**
Male, *n* (%)	121 (69.9%)	51 (63.8%)	0.403
Age, median (IQR)	61 (17)	72 (10.3)	<0.001^*^
**Comorbidities**			
Hypertension, *n* (%)	83 (47.9%)	44 (55%)	0.366
Diabetes, *n* (%)	49 (28.3%)	26 (32.5%)	0.597
Dyslipidemia, *n* (%)	73 (42.2%)	48 (60%)	0.012^*^
Obesity, *n* (%)	46 (26.6%)	17 (21.3%)	0.449
Cardiovascular disease, *n* (%)	16 (9.3%)	11 (13.8%)	0.390
**Chronic respiratory disease**, ***n*** **(%)**			
Asthma	8 (4.6%)	6 (7.5%)	0.008^*^
COPD	8 (4.6%)	9 (11.3%)	
Interstitial lung disease	1 (0.6%)	5 (6.3%)	
Bronchiectasis	1 (0.6%)	0	
OSAS	9 (5.2%)	6 (7.5%)	
History of malignancy, *n* (%)	20 (11.6%)	17 (21.3%)	0.066
Hepatopathy, *n* (%)	19 (10.9%)	3 (3.8%)	0.097
Chronic kidney disease, *n* (%)	11 (6.4%)	16 (20%)	0.002^*^
Immunosuppression, *n* (%)	6 (3.5%)	7 (8.8%)	0.121
**Symptoms**			
Dyspnea, *n* (%)	103 (59.5%)	46 (57.5%)	0.866
Cough, *n* (%)	134 (77.5%)	55 (68.8%)	0.185
Fever, *n* (%)	149 (86.1%)	62 (77.5%)	0.125
Myalgias, *n* (%)	53 (30.6%)	18 (22.5%)	0.235
Diarrhea, *n* (%)	45 (26%)	19 (23.8%)	0.819
Nausea, *n* (%)	14 (8.1%)	8 (10%)	0.794
Days from symptom onset to hospital admission), median (IQR)	8 (5)	8 (4.8)	0.457
Days from symptom onset to IMCU admission), median (IQR)	10 (7)	10 (7)	0.745
**Chest X-ray on admission**			
Bilateral opacities, *n* (%)	158 (92.9%)	74 (92.5%)	>0.999
Peripheral distribution, *n* (%)	105 (61.8%)	39 (48.8%)	0.071
**Laboratory blood tests**			
Leukocyte count (×10^9^/L), median (IQR)	8.20 (5.2)	10.20 (5.7)	<0.001^*^
Lymphocyte count (× 10^9^/L), median (IQR)	0.75 (0.6)	0.52 (0.5)	<0.001^*^
Lactate dehydrogenase (U/L), median (IQR)	395 (184.3)	476 (251)	<0.001^*^
C-Reactive protein (mg/L), median (IQR)	108 (167)	169 (196.5)	<0.001^*^
Ferritin (μg/L), median (IQR)	1,369 (1,213.5)	1,792 (1,444)	0.128
D-dimer (μg/L), median (IQR)	432 (784)	701 (1,756)	<0.001^*^
**Treatment**			
Lopinavir/Ritonavir, *n* (%)	134 (77.5%)	68 (85%)	0.222
Remdesivir, *n* (%)	9 (5.2%)	2 (2.5%)	0.510
Hydroxychloroquine, *n* (%)	164 (94.8%)	79 (98.8%)	0.177
Tocilizumab, *n* (%)	83 (47.9%)	41 (51.3%)	0.727
**Systemic corticosteroids**, ***n*** **(%)**			
Intravenous bolus	70 (40.5%)	44 (55%)	0.002^*^
Intravenous bolus + oral tapering regimen	46 (26.6%)	6 (7.5%)	
Oral tapering regimen	3 (1.7%)	1 (1.3%)	
**SpO2/FiO2 ratio, median (IQR)**	137.10 (82.2)	123.39 (37.4)	<0.001^*^
**Respiratory support**			
High-flow oxygen, *n* (%)	110 (63.6%)	55 (68.8%)	0.509
Non-invasive ventilation, *n* (%)	64 (36.9%)	69 (86.3%)	<0.001^*^
Invasive mechanical ventilation, *n* (%)	42 (24.3%)	40 (50%)	<0.001^*^
Tracheostomy, *n* (%)	18 (10.4%)	11 (13.8%)	0.572
**Transfer to ICU**, ***n*** **(%)**	51 (29.5%)	41 (51.3%)	0.001^*^
**Length of IMCU stay in days, median (IQR)**	6 (8)	4 (5)	<0.001^*^
**Length of hospital stay in days, median (IQR)**	18 (22)	12 (15)	<0.001^*^

*IQR, interquartile range; COPD, chronic obstructive pulmonary disease; OSAS, obstructive sleep apnea syndrome; SpO2, oxygen saturation; FiO2, fraction of inspired oxygen; IMCU, intermediate care unit; ICU, intensive care unit.*

* *Indicates significant p-value < 0.05*.

We compared groups of deceased patients. Subjects who died at the IMCU were significantly older (median 76 years, IQR 4.5 vs. median 66 years, IQR 12; *p* < 0.001). Nevertheless, similar proportions of gender, comorbidities and laboratory findings on admission were observed between groups.

### Survival Analysis

**Figure 2:**
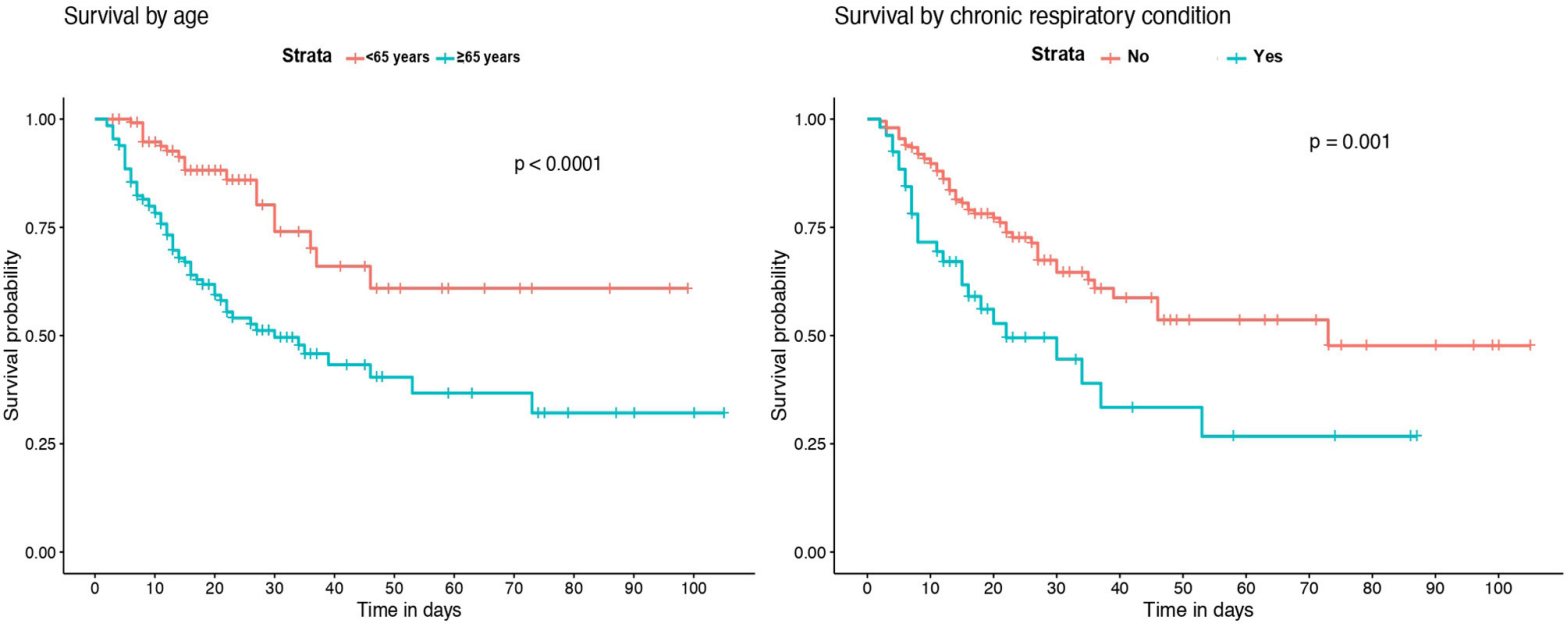
Kaplan–Meier survival analysis by age and previous chronic respiratory conditions.

**Figure 3:**
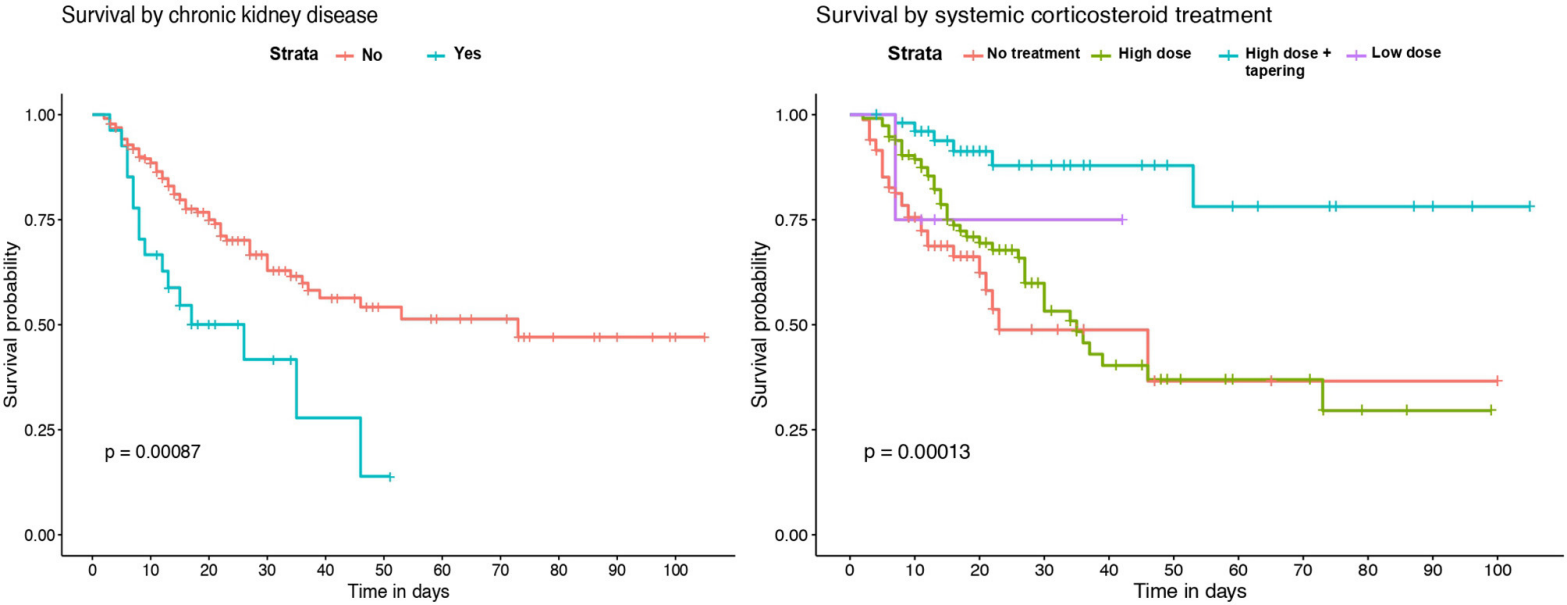
Kaplan–Meier survival analysis by previous chronic renal disease and systemic corticosteroid treatment.

Kaplan-Meier survival analysis identified a significant higher mortality in patients of 65 years of age or older (Figure 2). Also, significant differences in survival time were observed regarding chronic respiratory and renal conditions and corticosteroid treatment during hospitalization (Figure 3).

**Figure 4:**
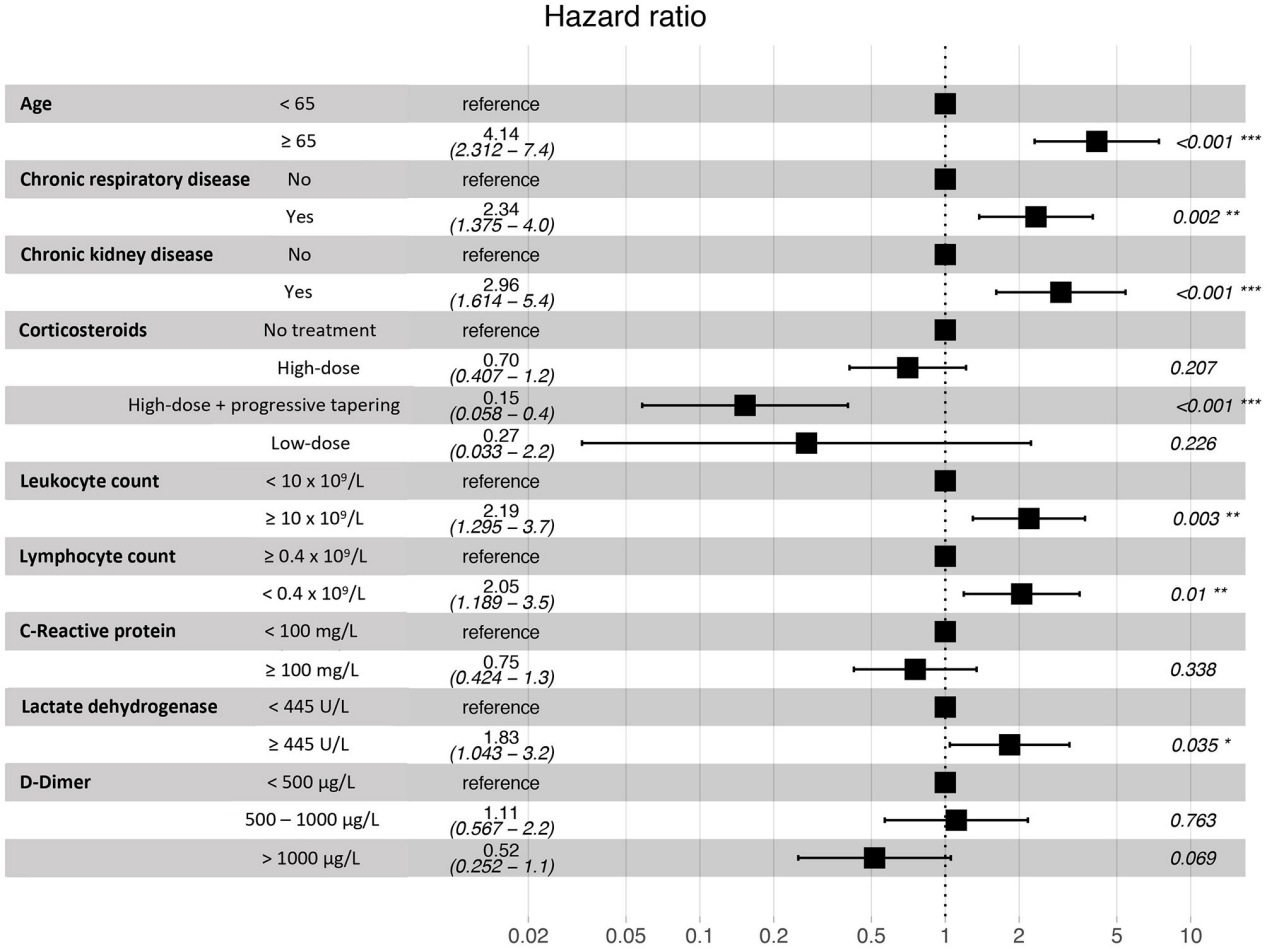
Multivariable Cox proportional hazards model for the assessment of in-hospital death risk.

Variables that independently increased risk of death in the multivariable Cox proportional hazards analysis included: age equal or older than 65 years [hazard ratio (HR) 4.14; 95% confidence interval (CI) 2.31–7.42; *p* < 0.001]; chronic respiratory conditions (HR 2.34; 95% CI 1.38–3.99; *p* = 0.002) and chronic kidney disease (HR 2.96; 95% CI 1.61–5.43; *p* < 0.001). Patients receiving high dose systemic corticosteroids followed by or progressive dose tapering showed a significant lower risk of death (HR 0.15; 95% CI 0.06–0.40; *p* < 0.001). Regarding laboratory findings on admission, blood leukocyte counts higher than 10 × 10^9^/L and lymphocyte counts lower than 0.4 × 10^9^/L were associated with higher risk of death (HR 2.19; 95% CI 1.30–3.71; *p* = 0.003 and HR 2.05; 95% CI 1.19–3.53; *p* = 0.010). Furthermore, serum LDH higher than 445 U/L was also independently associated with mortality (HR 1.83; 95% CI 1.04–3.21; *p* = 0.035). Figure 4 shows the results for the multivariable Cox proportional hazards model.

## Discussion

This is one of the first and largest studies to assess the clinical outcomes of severe COVID-19 patients admitted to an IMCU during the first wave of the pandemic. The IMCU allows a secure environment for providing non-invasive respiratory support and patient monitoring, potentially improving healthcare resource management. Our patient model management, including continuous assessment by specialists of respiratory medicine, critical care and internal medicine showed relatively low mortality rates and positive clinical outcomes compared to results from other critical care cohorts. Factors associated with higher mortality in these patients were older age, chronic pulmonary or kidney diseases, while treatment with systemic corticosteroid treatment was protective.

Patient characteristics and clinical presentation of the disease were similar to what has been described in previous studies (7, 18–20). Our cohort includes a large number of patients with severe respiratory failure, determined by a median SpO2/FiO2 ratio of 132.90. These patients may have required admission to ICU in hospitals without IMCU, possibly leading to further ICU collapse. To this date, there is limited data addressing the specific role of IMCU as a way of reducing the ICU transfer rate of severe COVID-19 patients. A study by Lagi et al. showed that improving nurse/patient ratio to 1:6 and using HFNC on regular wards resulted in a 12% reduction of ICU transfer (21). In this regard, our hospital rapidly increased the number of IMCU beds due to the pandemic situation, maintaining a nurse/patient ratio of 1:4 and non-invasive monitoring. In our cohort, only 36% of patients admitted to an IMCU required upscaling management to the ICU. This resulted in a reduction of ICU burden and allowed for more response time to face the rapid increase of severe cases. A previous study by Heili-Frades et al. showed that IMCU may avoid ~500.000 euros per year of hospital costs, especially in high complexity patients requiring HFNC oxygen therapy or NIV (8). Although the specific admission costs of COVID-19 patients have not been estimated, the IMCU not only could help to improve ICU bed availability, but also to lower overall healthcare costs.

All-cause mortality in our cohort was 31.6%, similar to what has been observed in other cohorts. A recent study by Li et al. showed that mortality in severe cases was 32.5% during the 32 days follow-up period, regardless of respiratory support requirement (18). Also, two cohorts of patients admitted to critical care (ICU or IMCU), one from UK and the other from Italy, reported a similar mortality rate (14). A multicenter European cohort study demonstrated that the availability of IMCU significantly reduced adjusted hospital mortality for adults admitted to the ICU (11). However, data is scarce regarding the mortality of severe COVID-19 patients specifically in an IMCU. In this regard, Franco et al. observed that the implementation of non-invasive respiratory support outside the ICU had favorable results, with an overall mortality rate of 26.9% (22). Similarly, we observed that mortality in IMCU patients who did not require ICU admission was 24.2%, significantly lower than in the ICU group. This may be expected, as most of the patients in the ICU group were more severely ill and required invasive mechanical ventilation. A large proportion of deceased patients received respiratory support with NIV, as this was the escalation support for those patients that continued to show clinical worsening with HFNC, unless otherwise decided by the patient and/or the multidisciplinary medical team. Although we cannot rule out the possible effect of delayed intubation on these results, all patients with NIV were assessed continuously by pulmonologists and intensive care physicians to determine whether intubation and IMV were required or not.

Survival analysis showed significant differences between patients of 65 years of age or older, and in those with chronic renal and respiratory diseases. These conditions were identified as independent risk factors for in-hospital mortality. Older age has been associated with an increase in the risk of death in several previous studies (18–20, 23). However, few of the published multivariable models for mortality risk in COVID-19 patients include chronic respiratory and renal diseases. Our results are in agreement with recent observations showing that patients with chronic obstructive pulmonary disease, interstitial lung diseases or chronic kidney disease that require hospitalization because of COVID-19 have higher risk of death (7, 20, 24). While the overall in-hospital mortality rate of interstitial lung disease (ILD) patients was 49% in the ISARIC4C study (24), the mortality rate of those ILD patients in our IMCU cohort was 83.3%, which suggests that the requirement of high-flux or non-invasive mechanical ventilation in ILD patients with severe COVID-19 associates a poor prognosis. Regarding laboratory findings, our model results show similarities with observations from prior cohorts, where patients with leukocytosis, lymphopenia and elevated serum LDH on admission have a higher mortality risk (4, 18, 19, 23).

Concerning patient treatment, only systemic corticosteroids were independently associated with a reduction of mortality in our cohort. Subjects receiving 3 days of high-dose methylprednisolone or dexamethasone followed by 7 days of oral dose tapering had a lower risk of death than those who received shorter treatments or were not treated. The positive effect of systemic corticosteroids has been described in recent studies. In a cohort from Wuhan, patients treated with methylprednisolone had a lower mortality rate (23). Also, a preliminary report of the RECOVERY trial showed that patients receiving dexamethasone for up to 10 days resulted in lower all-cause mortality (15). Furthermore, a recent meta-analysis by the World Health Organization Rapid Evidence Appraisal for COVID-19 Therapies (REACT) Working Group concluded that the administration of systemic corticosteroids in critically-ill COVID-19 patients was associated with a lower 28-day mortality, compared to usual care or placebo (25). Nevertheless, though the beneficial effect is clear in severe cases, the optimal dose and dose-reduction should be better evaluated for avoiding adverse events at the same time than achieving a proper lung recovery.

This study has several limitations, mainly related to the retrospective design of the analysis including a single center. The lack of a control group (non-IMCU hospital) does not allow to directly quantify the impact of IMCU in COVID-19 mortality or health-care burden. Also, our cohort included only severe patients, as we focused on the role of IMCU in patient management. This may limit the generalization of our results to less severe cases. Furthermore, local treatment protocols changed during the inclusion period due to the pandemic situation and the scarce data on COVID-19, which may have influenced the clinical outcomes of our study. However, the number of participants is higher than most previous studies, and our results agree with observations from different cohorts.

## Conclusions

Our study shows that IMCU may be useful tools for the multidisciplinary management of severe COVID-19 patients requiring non-invasive respiratory support and monitoring, therefore helping to reduce ICU burden. Older age and chronic respiratory or renal conditions are associated with worse clinical outcomes while treatment with systemic corticosteroids may have a protective effect on mortality. These results may help to further validate the feasibility of IMCU during the COVID-19 pandemic.

## Data Availability Statement

The raw data supporting the conclusions of this article will be made available by the authors, without undue reservation.

## Ethics Statement

The studies involving human participants were reviewed and approved by Bellvitge University Hospital Ethics Committee. Study reference No. PR260/20. Written informed consent for participation was not required for this study in accordance with the national legislation and the institutional requirements.

## Author Contributions

GS-C and MG participated in study design, acquisition, analysis, and interpretation of data, and in the elaboration of the manuscript. GB participated in data acquisition and in the elaboration of the manuscript. YR participated in data acquisition, analysis, and interpretation of data. MH-A, AM, PT-S, AC, EC, MS, AA, NR, and CS participated in data acquisition and analysis. JS participated in data acquisition, analysis, and interpretation of the results. MM-M participated in study design, interpretation of data, and in the elaboration of the manuscript. SS participated in study design, interpretation of data, and in the elaboration of the manuscript. All authors read and approved the final manuscript.

## Conflict of Interest

GS-C reports grants from Grifols. MM-M reports grants from Boehringer Ingelheim, Roche, Glaxo-Smith-Kline, Esteve-Teijin, Almirall, and Chiesi outside the submitted work. The remaining authors declare that the research was conducted in the absence of any commercial or financial relationships that could be construed as a potential conflict of interest.

## References

[1] Zhu N Zhang D Wang W Li X Yang B Song J. A novel coronavirus from patients with pneumonia in China, 2019. N Engl J Med. (2020) 382:727–33. 10.1056/NEJMoa2001017 31978945 PMC7092803

[2] Verity R Okell LC Dorigatti I Winskill P Whittaker C Imai N. Estimates of the severity of coronavirus disease 2019: a model-based analysis. Lancet Infect Dis. (2020) 20:669–77. 10.1016/S1473-3099(20)30243-7 32240634 PMC7158570

[3] Cinesi Gómez C Peñuelas Rodríguez Ó Luján Torné M Egea Santaolalla C Masa Jiménez JF García Fernández J. Clinical consensus recommendations regarding non-invasive respiratory support in the adult patient with acute respiratory failure secondary to SARS-CoV-2 infection. Arch Bronconeumol. (2020) 56 (Suppl 2):11–8. 10.1016/j.redare.2020.05.001 34629620 PMC7270645

[4] Zhou F Yu T Du R Fan G Liu Y Liu Z. Clinical course and risk factors for mortality of adult inpatients with COVID-19 in Wuhan, China: a retrospective cohort study. Lancet. (2020) 395:1054–62. 10.1016/S0140-6736(20)30566-3 32171076 PMC7270627

[5] Wang D Hu B Hu C Zhu F Liu X Zhang J. Clinical characteristics of 138 hospitalized patients with 2019 novel coronavirus-infected pneumonia in Wuhan, China. JAMA. (2020) 323:1061–9. 10.1001/jama.2020.1585 32031570 PMC7042881

[6] Huang C Wang Y Li X Ren L Zhao J Hu Y. Clinical features of patients infected with 2019 novel coronavirus in Wuhan, China. Lancet. (2020) 395:497–506. 10.1016/S0140-6736(20)30183-5 31986264 PMC7159299

[7] Docherty AB Harrison EM Green CA Hardwick HE Pius R Norman L. Features of 20 133 UK patients in hospital with covid-19 using the ISARIC WHO Clinical Characterisation Protocol: prospective observational cohort study. BMJ. (2020) 369:m1985. 10.1136/bmj.m1985 32444460 PMC7243036

[8] Heili Frades S Carballosa de Miguel M del P Naya Prieto A Galdeano Lozano M Mate García X Mahillo Fernández I. Cost and mortality analysis of an intermediate respiratory care unit. is it really efficient and safe? Arch Bronconeumol. (2019) 55:634–41. 10.1016/j.arbr.2019.06.008 31587917

[9] Prin M Wunsch H. The role of stepdown beds in hospital care. Am J Respir Crit Care Med. (2014) 190:1210–6. 10.1164/rccm.201406-1117PP 25163008 PMC4315815

[10] Plate JDJ Leenen LPH Houwert M Hietbrink F. Utilisation of intermediate care units: a systematic review. Crit Care Res Pract. (2017) 2017:8038460. 10.1155/2017/8038460 28775898 PMC5523340

[11] Capuzzo M Volta CA Tassinati T Moreno RP Valentin A Guidet B. Hospital mortality of adults admitted to intensive care units in hospitals with and without intermediate care units: a multicentre European cohort study. Crit Care. (2014) 18:551. 10.1186/s13054-014-0551-8 25664865 PMC4261690

[12] European Centre for Disease Prevention and Control. COVID-19 situation update worldwide, as of May 12, 2021. Available online at: https://www.ecdc.europa.eu/en/geographical-distribution-2019-ncov-cases (accessed May 17, 2021).

[13] Chen N Zhou M Dong X Qu J Gong F Han Y. Epidemiological and clinical characteristics of 99 cases of 2019 novel coronavirus pneumonia in Wuhan, China: a descriptive study. Lancet. (2020) 395:507–13. 10.1016/S0140-6736(20)30211-7 32007143 PMC7135076

[14] Grasselli G Zangrillo A Zanella A Antonelli M Cabrini L Castelli A. Baseline Characteristics and Outcomes of 1,591 Patients Infected with SARS-CoV-2 Admitted to ICUs of the Lombardy Region, Italy. JAMA. (2020) 323:1574–81. 10.1001/jama.2020.5394 32250385 PMC7136855

[15] The RECOVERY Collaborative Group. Dexamethasone in hospitalized patients with covid-19—preliminary report. N Engl J Med. (2020) 384:693–704. 10.1056/NEJMoa2021436 32678530 PMC7383595

[16] Rice TW Wheeler AP Bernard GR Hayden DL Schoenfeld DA Ware LB. Comparison of the SpO2/FIO2 ratio and the PaO2/FIO2 ratio in patients with acute lung injury or ARDS. Chest. (2007) 132:410–7. 10.1378/chest.07-0617 17573487

[17] Guan W Ni Z Hu YY Liang W Ou C He J. Clinical characteristics of coronavirus disease 2019 in China. N Engl J Med. (2020) 382:1708–20. 10.1056/NEJMoa2002032 32109013 PMC7092819

[18] Li X Xu S Yu M Wang K Tao Y Zhou Y. Risk factors for severity and mortality in adult COVID-19 inpatients in Wuhan. J Allergy Clin Immunol. (2020) 146:110–8. 10.1016/j.jaci.2020.04.006 32294485 PMC7152876

[19] Du RH Liang LR Yang CQ Wang W Cao TZ Li M. Predictors of mortality for patients with COVID-19 pneumonia caused by SARSCoV-2: a prospective cohort study. Eur Respir J. (2020) 55:2000524. 10.1183/13993003.00524-2020 32269088 PMC7144257

[20] Imam Z Odish F Gill I O'Connor D Armstrong J Vanood A. Older age and comorbidity are independent mortality predictors in a large cohort of 1,305 COVID-19 patients in Michigan, United States. J Intern Med. (2020) 288:469–76. 10.1111/joim.13119 32498135 PMC7300881

[21] Lagi F Piccica M Graziani L Vellere I Botta A Tilli M. Early experience of an infectious and tropical diseases unit during the coronavirus disease (COVID-19) pandemic, Florence, Italy, February to March 2020. Eurosurveillance. (2020) 25:2000556. 10.2807/1560-7917.ES.2020.25.17.2000556 32372754 PMC7201949

[22] Franco C Facciolongo N Tonelli R Dongilli R Vianello A Pisani L. Feasibility and clinical impact of out-of-ICU non-invasive respiratory support in patients with COVID-19 related pneumonia. Eur Respir J. (2020) 56:2002130. 10.1183/13993003.02130-2020 32747398 PMC7397952

[23] Wu C Chen X Cai Y Xia J Zhou X Xu S. Risk factors associated with acute respiratory distress syndrome and death in patients with coronavirus disease 2019 pneumonia in Wuhan, China. JAMA Intern Med. (2020) 180:934–3. 10.1001/jamainternmed.2020.0994 32167524 PMC7070509

[24] Drake TM Docherty AB Harrison EM Quint JK Adamali H Agnew S. Outcome of hospitalization for covid-19 in patients with interstitial lung disease: an international multicenter study. Am J Respir Crit Care Med. (2020) 202:1656–65. 10.1164/rccm.202007-2794OC 33007173 PMC7737581

[25] WHO Rapid Evidence Appraisal for COVID-19 Therapies (REACT) Working Group Sterne JAC Murthy S Diaz JV Slutsky AS Villar J Angus DC. Association between administration of systemic corticosteroids and mortality among critically ill patients with covid-19: a meta-analysis. JAMA. (2020) 324:1330–41. 10.1001/jama.2020.17023 32876694 PMC7489434

